# The GH-IGF-SST system in hepatocellular carcinoma: biological and molecular pathogenetic mechanisms and therapeutic targets

**DOI:** 10.1186/1750-9378-9-27

**Published:** 2014-08-20

**Authors:** Claudia Pivonello, Maria Cristina De Martino, Mariarosaria Negri, Gaia Cuomo, Federica Cariati, Francesco Izzo, Annamaria Colao, Rosario Pivonello

**Affiliations:** 1 Dipartimento di Medicina Clinica e Chirurgia, Università Federico II di Napoli, Via Sergio Pansini, 5, Naples 80131, Italy; 2 IRCCS Fondazione SDN, Naples, Italy; 3 National Cancer Institute G Pascale Foundation, Naples, Italy

**Keywords:** GH-IGF1 axis, Somatostatin, Somatostatin receptors, Normal liver, Hepatocarcinogenesis, Hepatocarcinoma

## Abstract

Hepatocellular carcinoma (HCC) is the sixth most common malignancy worldwide. Different signalling pathways have been identified to be implicated in the pathogenesis of HCC; among these, GH, IGF and somatostatin (SST) pathways have emerged as some of the major pathways implicated in the development of HCC. Physiologically, GH-IGF-SST system plays a crucial role in liver growth and development since GH induces IGF1 and IGF2 secretion and the expression of their receptors, involved in hepatocytes cell proliferation, differentiation and metabolism. On the other hand, somatostatin receptors (SSTRs) are exclusively present on the biliary tract. Importantly, the GH-IGF-SST system components have been indicated as regulators of hepatocarcinogenesis. Reduction of GH binding affinity to GH receptor, decreased serum IGF1 and increased serum IGF2 production, overexpression of IGF1 receptor, loss of function of IGF2 receptor and appearance of SSTRs are frequently observed in human HCC. In particular, recently, many studies have evaluated the correlation between increased levels of IGF1 receptors and liver diseases and the oncogenic role of IGF2 and its involvement in angiogenesis, migration and, consequently, in tumour progression. SST directly or indirectly influences tumour growth and development through the inhibition of cell proliferation and secretion and induction of apoptosis, even though SST role in hepatocarcinogenesis is still opened to argument.

This review addresses the present evidences suggesting a role of the GH-IGF-SST system in the development and progression of HCC, and describes the therapeutic perspectives, based on the targeting of GH-IGF-SST system, which have been hypothesised and experimented in HCC.

## Introduction

Hepatocellular carcinoma (HCC) represents the sixth most common cancer and the third leading cause of mortality for malignancy in the world [1]. HCC is the predominant type of hepatic carcinoma, accounting for 90% of liver primary malignant tumours [1-3]. The Barcelona Clınic Liver Cancer (BCLC) staging system is recommended by European Association for the Study of the Liver (EASL) and the European Organisation for Research and Treatment of Cancer (EORTC) for prognostic prediction and treatment allocation of HCC [1,4,5]. BCLC staging classification is based on variables related to tumour stage (number and diameter of tumour nodules and presence of portal invasion and metastasis), liver function (Child-Pugh class) and general health status (performance status test), that define five groups of patients with HCC (0, A, B, C and D) and link these groups to a specific treatment strategy and a different prognosis [1,4,5]. In particular, according to BCLC staging system, the “stage 0” includes patients with very early HCC; the “stage A” patients with early HCC; the “stage B” patients with intermediate HCC; the “stage C” patients with advanced HCC and the “stage D” patients with endstage disease [1,4,5].

**Figure 1:**
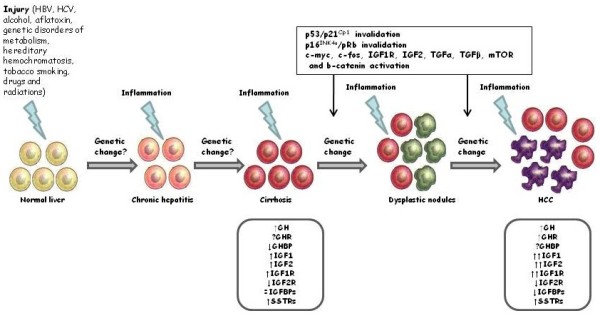
**Scheme of hepatocarcinogenesis and relation with GH-IGF-SST system.** Hepatocarcinogenesis is a multistep process generally evolving from normal liver through chronic hepatitis, cirrhosis and formation of dysplastic nodules to HCC. Several hepatocarcinogens (virus infection, alcohol consumption, genetic disorders of metabolism, hereditary hemochromatosis, tobacco smoking, aflatoxins, drugs and radiations) could cause direct or indirect damage to hepatocytes DNA (genetic change?), thus inducing a hyperproliferative status and inflammatory reactions, in particular during chronic exposure. The persistent liver damage can lead to the activation and/or inhibition of several molecular cell pathways, predisposing to the accumulation of genetic and epigenetic alterations that determines HCC development. The most important changes of molecular pathways described in HCC are reported in the text box on top of the figure. The most important changes in GH-IGF-SST system occurring during the different steps of HCC development are reported in the text boxes on the bottom of the figure.

Hepatocarcinogenesis is a multistep process evolving from normal liver, through chronic hepatitis and/or cirrhosis and formation of dysplastic nodules, to HCC. Indeed, HCC rarely arises in non-cirrhotic liver (about 20%), while it often develops on pre-existing cirrhosis (about 80%), primarily due to hepatitis B virus (HBV) or C virus (HCV) infection or alcohol consumption. The aetiological factors (virus infection, alcohol consumption, genetic disorders of metabolism, hereditary hemochromatosis, tobacco smoking, aflatoxins, drugs and radiations) may vary depending on ethnic group or geographic area of the patients affected by this malignancy [1,6]. These evidences suggest that several pathological agents, able to induce liver damage, can stimulate an inflammatory and hyperproliferative response in hepatocytes by activating and/or inhibiting several cell molecular pathways. Liver damage, chronic inflammation and the hyperproliferative hepatocytes status predispose to the accumulation of genetic and epigenetic alterations that determine the development of HCC [6-15]. The types and the sequence of the occurrence of genetic and molecular alterations associated with hepatocarcinogenesis have not been fully clarified and they seem to be highly tumour-specific [16]. The commonly altered pathways in HCC include: IGF (in particular IGF2 and downstream mediators, as mTOR), p16/pRb (DNA repair pathway), p53/p21 (cell-cycle pathway), β-catenin, and trasforming growth factor (TGF) [6-18]. A schematic model of hepatocarcinogenesis is presented in Figure 1.

The GH-IGF-SST system is an endocrine system consisting of growth hormone (GH), insulin-like growth factors (IGF1 and IGF2) and the relative associated carrier proteins and receptors, and somatostatin (SST), controlling human prenatal development and postnatal growth by regulating cell proliferation, differentiation and metabolism [19]. The liver occupies a central role in this endocrine system, because it produces many of its components and it is also a target of their actions [20]. Therefore, liver injury affects the function of GH-IGF-SST system and, in turn, the alteration of GH-IGF-SST system may play a role in the development of liver diseases, such as cirrhosis, fibrosis and HCC [21].

The GH-IGF-SST system seems to play a role in the development of various malignancies, including HCC. Indeed, in several types of cancer, GH-IGF1 axis has been demonstrated to affect tumour cell proliferation, apoptosis and invasiveness, and tumour angiogenesis [22,23]. Changes in the expression pattern of GH-IGF axis have been reported in HCC, suggesting that this system plays a role in hepatocarcinogenesis [24]. Additionally, the activation of somatostatin receptors (SSTRs) may elicitate antitumoural effects through both direct (inhibition of cell proliferation and induction of apoptosis) and indirect (inhibition of cell proliferation and angiogenesis through the suppression of growth factors and growth-promoting hormones, such as GH and IGF1) mechanisms [25,26]. HCC has been reported to express SSTRs, although literature data about the antineoplastic effects of the somatostatin analogues (SA) in HCC are still controversial [27,28].

The aim of this review is to analyse the role of GH-IGF-SST system in the development of HCC, mainly focusing on the underlying biological and molecular mechanisms and on the possibility to target this pathway as a new treatment strategy in HCC patients.

### GH-IGF-SST system: an outline

The GH-IGF-SST system is composed by three essential components: ligands (GH, IGF1 and IGF2, SST), receptors [GH receptor (GHR), IGF1 receptor (IGF1R), IGF2 receptor (IGF2R) and SSTRs)] and binding proteins [GH binding protein (GHBP) and IGF binding proteins (IGFBPs)].

GH is mainly produced and secreted in a pulsatile manner by the anterior pituitary gland and represents the main regulator of postnatal growth, by controlling cell secretion, metabolism, survival and proliferation [29,30]. GH promotes IGF1 gene transcription and synthesis in the liver, thus regulating the circulating levels of IGF1 [31].

**Figure 2:**
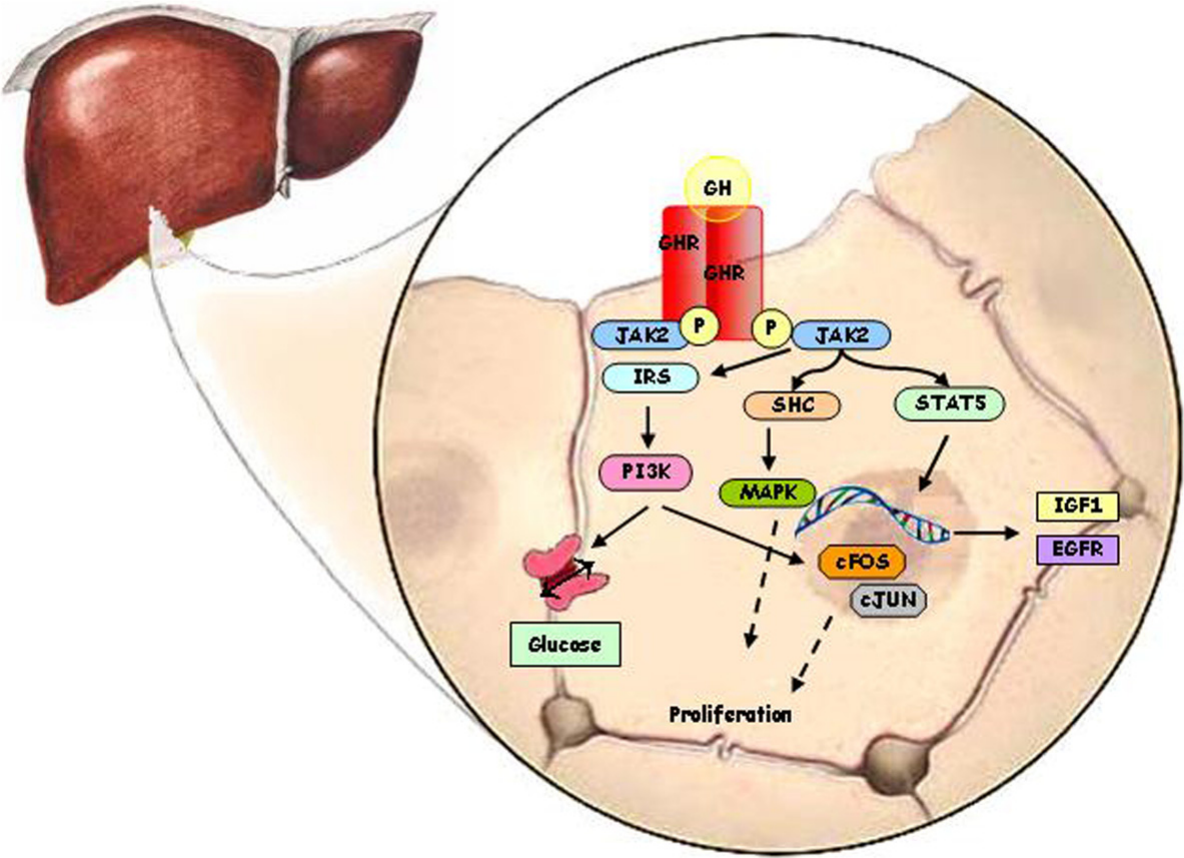
**Intracellular pathway associated with GHR activation in hepatocytes.** GH exerts its effect by binding to the extracellular domain of the GHR, where one molecule of GH binds two GHRs increasing the affinity of both receptors for two molecules of JAK2, which phosphorylate the GHR. GHR activation, in turn, triggers the activation of several signal transduction pathways, including STAT and PI3K through IRS. IRS and PI3K can activate nuclear transcription factors, including c-FOS and c-JUN, to induce cell proliferation and differentiation but also can increase glucose transport. GH-induced JAK2 activation phosphorilates STAT5 which translocates into the nucleus where binds to response elements in the regulatory regions of target genes including IGF1 and EGFR. The activation of adaptor protein SHC leads to the activation of MAPK involved in cell proliferation and growth.

GH secretion is strictly controlled by the hypothalamic neuropeptides growth hormone releasing hormone (GHRH) and SST. GHRH is produced in the arcuate nucleus of the hypothalamus and it represents the central stimulator and regulator of GH synthesis and release. SST is produced in the periventricular nucleus of the hypothalamus and it mediates the negative feedback operated by GH on its own release, by acting through seven transmembrane domain G-protein-coupled receptors, the SSTRs subtypes 1–5 [32]. SSTR2 has been reported to be the dominant SSTR influencing GH release from the somatotroph cells; the inhibition of Ca^2+^ influx (through L- and T-type voltage-sensitive channels), the stimulation of K^+^ influx and the inhibition of cAMP levels have been reported to be the dominant signal transduction involved in this SSTR2 function [33]. GH action is mediated *via* GHR, which is widely expressed in many human tissues. GHR exists as pre-formed dimers; conformational changes induced by ligand binding activate signal transduction [34]. GH/GHR dimer interaction mainly results in the activation of different tyrosine kinases. The activation of JAK2, a protein of Janus kinase family, is thought to be the key regulator of GH transduction signalling. Several signalling proteins and downstream pathways are activated as a consequence of GHR/JAK2 complex formation, including STAT (signal transducers and activators of transcription) 1, 2, 3 and 5 (mainly 5a and 5b), phosphatidylinositol 3-kinases (PI3K) and mitogen-activated protein kinases (MAPKs) [34]. STAT5a and STAT5b activation is critical for some important GH functions, including the regulation of body growth and metabolism, and, in particular, the stimulation of IGF1 synthesis [35] and the regulation of the expression of crucial liver genes [30,36]. Figure 2 shows a simplified scheme of the GH-activated intracellular pathways. GHBPs are produced by a proteolytic cleavage of GHR at the site proximal to the cell surface. GHBPs bind about half of the circulating GH and have several and complex functions, including the modulation of plasma GH half-life and the binding of GH to GHR [37].

**Figure 3:**
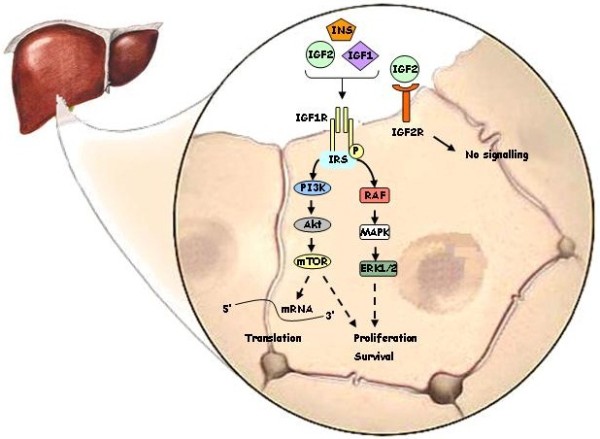
**Intracellular pathway associated with IGF1R activation.** IGF1R activation by INS, IGF1 and IGF2, leads to autophosphorylation on tyrosines 1131, 1135 and 1136 in the kinase domain, followed by recruitment of specific docking intermediates, such as members of the IRS family (IRS-1, IRS-2, IRS-3, IRS-4). This molecule link the IGF1R to diverse signalling pathways, allowing the induction of growth, transformation, differentiation and protection against apoptosis, primarily through the activation of the PI3K/Akt/mTOR and the Ras GTPase/Raf-1 (Raf)/Mek (MAPK)/Erk (ERK1/2) signalling pathways.

IGF1 plays an essential role in the body growth and metabolism, especially during the postnatal life, through the activation of IGF1R. It has been suggested that serum IGF1 postnatally supplied by the liver plays an endocrine role that is nearly as significant for the growth as the autocrine/paracrine action of IGF1 produced locally in various tissues [33]. On the other side, serum IGF1 exerts a negative feedback on GH production, by directly inhibiting the pituitary gland secretion and indirectly stimulating SST and inhibiting GHRH secretion [33]. IGF2 is a circulating peptide hormone whose regulation can be under the control of GH [38]. IGF2 plays an important role in the body growth and metabolism, especially during the prenatal life. Indeed, IGF2 is preferentially expressed during embryogenesis and foetal development, it stimulates cell growth and proliferation and it promotes embryo and fetus growth, by activating IGF1R and insulin receptor (IR), in particular the isoform A (IRA), which is predominantly expressed during prenatal life [39,40]. IGF2 can also bind to IGF2R, which is considered a “scavenger receptor”. Indeed, the binding of IGF2 to this receptor assigns IGF2 to degradation towards the lysosomes and does not elicit any proliferation or survival signals [41,42]. Overall, IGFs may also play an autocrine or paracrine role by binding to IGF1R and/or IR on target cells [43,44]. IGFs are involved in many cell processes, including cell differentiation, cell growth and proliferation, and apoptosis. These effects are predominantly mediated by the activation of two signalling cascades: MAPK and PI3K pathways. The binding of IGFs to their receptor tyrosine kinases (RTKs) triggers the phosphorylation of several substrates, including insulin receptor substrate (IRS1-4) proteins, leading to the activation of PI3K and, subsequentially, to the activation of the serine-threonine kinase (AKT) and downstream signalling effectors, including the mammalian target of rapamycin (mTOR), 4E-binding proteins (EIF4EBP1) and p70 ribosomal protein S6 kinase (RPS6KB1). In addition, IRS1 and IRS2 can activate rat sarcoma viral oncogene homolog (RAS) and, subsequentially, the MAPK pathway, including mitogen/extracellular signal-regulated kinase (MEK) and extracellular signal-regulated kinase (ERK) [44]. Figure 3 shows a simplified scheme of the IGF-activated intracellular pathways.

**Figure 4:**
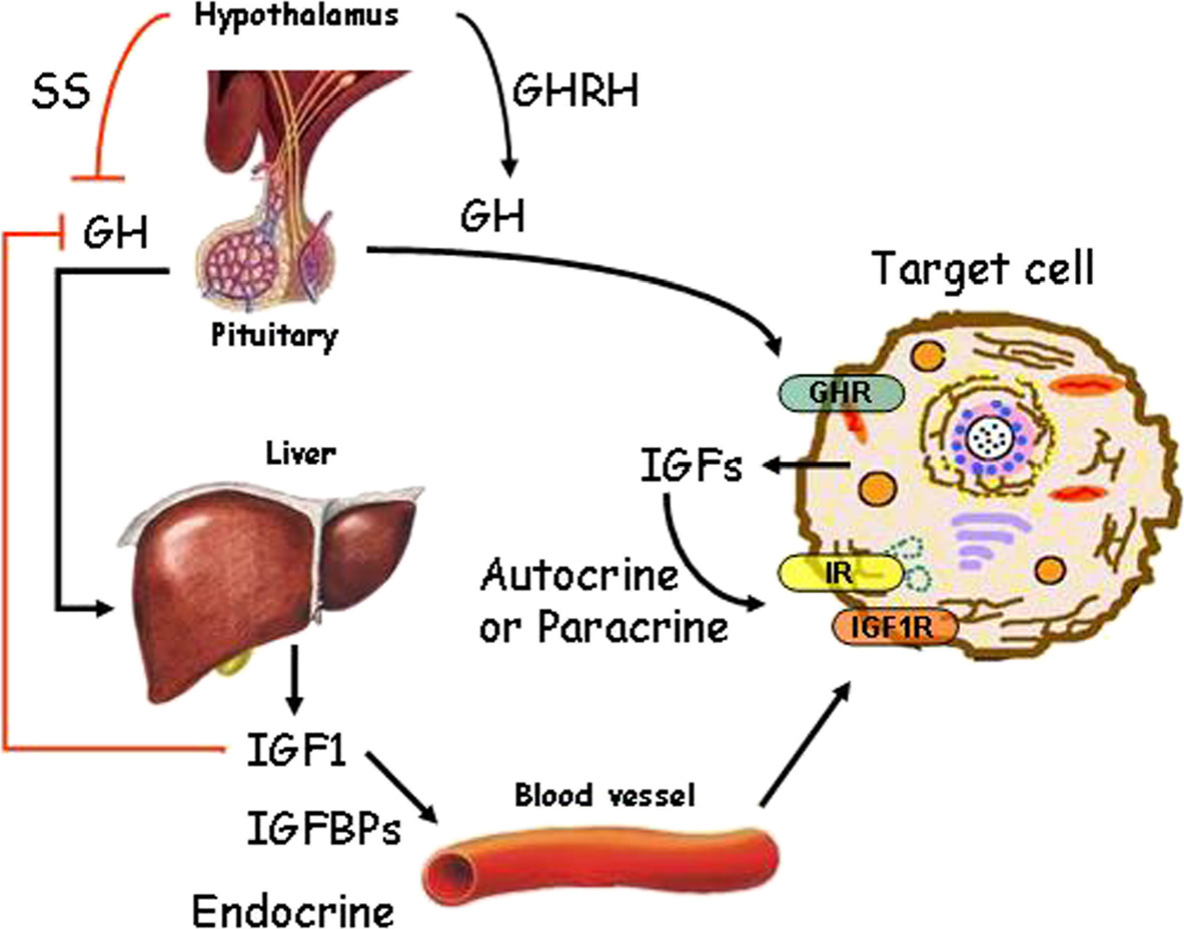
**Outline of endocrine, autocrine and paracrine GH-IGF-SST system in human adult life.** At the whole organism level, circulating IGF1 is mainly produced in the liver and IGF1 levels are under the control of GH. In turn, GH is regulated by SST and GHRH. IGF1 is usually delivered by circulation but it can also be produced in an autocrine or paracrine manner.

The IGFBP superfamily includes six proteins (IGFBP1-6), which are able to bind the IGFs with different affinity. IGFBP-IGF complexes influence IGFs activities by modulating their half-life and tissues bioavailability [45]. IGFBPs are produced by a variety of different tissues: IGFBP1 expression is restricted to the liver, IGFBP2 expression is predominat in tissues derived from ectoderm and endoderm, IGFBP3 and IGFBP4 are expressed in a specific subset of mesenchymal cells and IGFBP5 is expressed in tissues derived from ectoderm and muscle precursor cells [45]. IGFBPs are considered pleiotropic molecules, with IGF-dependent and IGF-independent actions. About 80% of circulating IGF1 is bound to IGFBPs, mainly to IGFBP3. IGFBP3 is the most abundant binding protein and binds IGF1 or IGF2 and the acid-labile subunit (ALS) protein, forming a ternary complex [43,44]. ALS is a glycoprotein that interacts with IGFBP3 only when IGFBP3 is associated to IGF1 or IGF2 [43,44]. This phenomenon is possible because, in normal conditions, the total IGFs and IGFBP3 are present in the serum at equimolar concentrations. To a smaller degree, IGFBP5 also forms a ternary complex with IGFs and ALS [45,46]. ALS increases the molecular mass of the IGF/IGFBP3 complex, by modulating the amount of IGFs that can diffuse to the extracellular fluids [45]. The other IGFBPs with low molecular mass do not form a ternary complex with any other protein, and mainly circulate associated in a binary complex with IGF1 or IGF2 [45,46]. IGF-independent actions of IGFBPs include effects on cell proliferation, apoptosis, motility and migration and they can be mediated by either intact IGFBPs or by their proteolysed fragments [47,48]. A schematic outline of GH-IGF-SST system in human adult life is represented in Figure 4.

### The GH-IGF-SST system: a focus on liver

The liver is the major target organ of GH, since GH induces IGF1 expression and secretion in hepatocytes, the liver parenchymal cells and, at a lesser extent, in cholangiocytes [49]. Moreover, in hepatocytes, GH is also deputed to the regulation of glucose metabolism by reducing glucose uptake and promoting gluconeogenesis [50].

IGF1 and IGF2 have broad insulin-like actions, including promotion of energy storage and fatty acid synthesis in the liver. In mice and humans, the circulating IGF1 derives predominantly from hepatocytes [51,52]. The IGF1 derived from the liver has a central role as mediator of GH in the regulation of postnatal growth. Indeed, studies conducted in bitransgenic mice with IGF1 gene expression in the liver but not in extrahepatic tissues, have demonstrated that the growth rate of these mutated mice was not significantly different from wild-type animals, suggesting that IGF1 derived from the liver is important but not essential for the postnatal growth [53]. IGF1 produced in extrahepatic tissues also plays an important role as autocrine and/or paracrine regulator of postnatal growth. Indeed, studies conducted in knockout mice, with liver-specific deletion of IGF1 gene and normal IGF1 gene in extrahepatic tissues, demonstrated that the growth rate of these mutated mice was not significantly different from wild-type animals, thus the IGF1 produced in extrahepatic tissues is important but not essential for the postnatal growth [54,55]. Therefore, both IGF1 produced in the liver and IGF1 produced in extrahepatic tissues play an important role in the postnatal growth, in both an endocrine and autocrine/paracrine manner [53-55].

IGF2 expression has been demonstrated in rat hepatocytes, Kupffer cells (KCs), hepatic endothelial cells (ECs) and hepatic stellate cells (HSCs) [56,57], and in human foetal and adult hepatocytes [52,58]. IGF2 gene transcription largely varies during development, depending on the activation of distinct patterns of four different IGF2 gene promoters (P1-P4). In rodents, promoters P2, P3 and P4 are active during foetal growth in the liver, whereas, after birth, P1 becomes the dominant promoter and P2-P4 decrease their activity [59]. In humans, the transcription of IGF2 during foetal development is preferentially under the regulation of P2, P3, P4 promoters, while all promoters regulate IGF2 transcription from two months after birth onwards [58].

IGF1R has been reported to be abudantly expressed in rat KCs, ECs and HSCs, whereas its expression in rat hepatocytes is scant, suggesting that IGF1 does not have a strong autocrine/paracrine direct effect on hepatocytes in this animal model [57,60]. IGF2R is expressed in rat non-parenchimal liver cells [57]. No studies reported IGF1R and IGF2R expression in human normal liver. IGFBP1, IGFBP2, IGFBP4 and ALS are produced by rat hepatocytes [61-63], whereas IGFBP3 seems to be produced by rat KCs, ECs and HSC [64,65]. In humans, messenger and protein expression of IGFBPs has been evaluated in the liver. IGFBP1, IGFBP2 and IGFBP4 messenger and protein are widely expressed in the parenchymal cell population; IGFBP3 messenger and protein are localized in KCs [52,66]; IGFBP5 is produced by HSCs [67]. These binding proteins, involved in the control of the pool of bioactive IGFs, are under the control of the nutritional status and well correlate with the liver functional reserve [68]. Several studies demonstrated that insulin, glucagon and IGFs are able to regulate the transcription of IGFBPs genes [65,68,69].

No studies reported SSTRs expression in mouse or rat normal liver. SSTRs expression has been evaluated in a human normal hepatocytes cell line, the L-02, in which SSTR2 and SSTR4 messengers have been detected by molecular techniques [70]. However, SSTRs protein expression has been reported in human normal liver tissue, at the level of cholangiocytes, but not in hepatocytes [27,71]. The main action of SST in the liver has been explored in pre-clinical *in vivo* studies. In rats subjected to bile duct ligation, SST affects cholangiocytes choleretic activity, by counteracting the effect of secretin on the biliary excretion of water and bicarbonate by cholangiocytes, in cholestatic conditions [72]. Moreover, studies performed in rats have demostrated that SST has a role in the regulation of lipid metabolism [73].

### The GH-IGF-SST system in hepatocarcinogenesis

A large number of studies has evaluated the role of GH-IGF-SST system in the development and progression of HCC, but results are controversial (Figure 1).

### GH and GH receptor

The role of GH on the development and progression of HCC is still matter of controversies.

In a preclinical setting, the use of recombinant human growth hormone (rhGH) has been tested predominantly in *in vitro* studies conducted in two HCC cell lines: Bel-7402 and SMMC-7721 [74]. Among these cell lines, only Bel-7402 cells were found to express GHR messenger [74]. The treatment with rhGH induced an increase of the percentage of cells in mitotic phase G2-M, as well as an increase of cell invasion and proliferation in Bel-7402, but not in SMMC-772 [74]. In addition, rhGH treatment induced an overexpression of vascular endothelial growth factor (VEGF) in Bel-7402 cells, but not in SMMC-7721 [74]. Based on this preclinical study, it can be speculated that rhGH could affect HCC growth in GHR positive HCC, both directly and indirectly, *via* VEGF secretion. The binding of GH to GHR triggers the activation of intracellular signals, among which the predominant effector is Stat5 [30]. Although the activation of Stat5 has been associated with tumourigenesis of several solid tumours [75], in the liver, Stat5 has a role as tumour promoter but it seem to have also a potential role as tumour suppressor gene [75,76]; therefore, the role of Stat5 in hepatocarcinogenesis is still debated. Stat5b enhances HCC cell aggressiveness through the induction of epithelial-mesenchymal transition, as demonstrated in a human transfected HCC cell line [77]. In apparent contrast, loss of STAT5 in mice caused liver steatosis and fibrosis, and promoted chemically induced HCC, by up-regulating the TGF-beta (TGF-β) and by altering the expression of cell cycle pathway regulators [76,78]. Liver steatosis results from excessive synthesis of free fatty acid, whose release is regulated not only by GH bu also by glucocorticoids (GCs). Recently, in a study investigating the role of combined deletion of hepatic STAT5 and glucocorticoid receptor (GR) in mice, it has been observed that the block of GH and GCs signallings induces lipid accumulation in the liver, which, in turn, contributes to liver chronic inflammation with progression to cirrhosis; this condition subsequently promotes tumorigenic transformation of hepatocytes [79]. This study suggests that hepatic GH signalling is crucial for the maintenance of lipid homeostasis and that the impairment of this signalling causes severe metabolic liver disease predisposing to HCC.

Few studies reporting GHR expression in HCC are presently available in literature [80-82]. In one of these studies, GHR expression has been investigated in 40 different samples of HCC tissues: GHR was undetectable in 5 samples, whereas in the remaining 35 HCC tissues GHR was expressed but it was associated with a lower GH binding ability compared to normal liver [82], suggesting that HCC could be partially resistent to GH stimulation. In HCC patients undergoing hepatectomy, rhGH has been evaluated as a potential treatment to improve the protein catabolism that complicates major surgical procedures. In this study, 24 HCC patients were randomly assigned to two postoperative treatment groups: parental nutrition *vs* parental nutrition plus rhGH. In the last group an increase of GH-IGF1 axis (evaluated as circulating IGF1 and IGFBP3) was observed without any change in tumour-free survival rates and median tumour-free survival time [83]. Chronic liver diseases were found to be associated with increased circulating GH levels, compared with healthy subjects [84,85]. However, in patients with cirrhosis, despite the increase in circulating GH levels, serum IGF1, IGFBP3 and ALS levels and the IGF1 response to GH were found to be lower than in controls, resembling a condition of GH resistance, as a consequence of liver dysfunction [86].

In summary, *in vitro* data suggest that GH can stimulate human HCC cell proliferation but patients with HCC seem to have a condition of GH resistence that might reduce these effects in an *in vivo* setting Further studies are still necessary to better address the role of GH in hepatocarcinogenesis.

### IGF1 and IGF2

The role of IGF1 and IGF2 on the development and progression of HCC has been widely studied.

IGF1 and IGF2 messenger expression in human HCC cell lines is reported in literature with conflicting results. Personal unpublished data seem to suggest that HepG2 and HuH-7 cell lines do not express IGF1 but largely express IGF2 [87]. The high expression of IGF2 messenger has been also reported in different studies [88,89], while endogenous IGF1 messenger expression has been reported in HepG2 and HuH-7 cell lines only in one study [90].

The role of IGF1 in hepatocarcinogenesis has been explored in preclinical settings, focusing on the ability of IGF1 to regulate HCV infection. HCV RNA-containing viruses are present in blood as hybrid particles, termed lipoviroparticles (LVPs), composed by host lipoproteins (low-density lipoprotein [LDL] and very-low-density lipoprotein [VLDL]) and immunoglobulins. As part of these LVPs, HCV are highly able to infect host cells by binding lipoprotein receptors and escaping antibodies recognition [91]. Recently, it has been demonstrated that the lipolytic enzyme lipoprotein lipase (LPL) inhibits HCV infection by blocking virus cell entry in a HCC cell line [92]. In a human HCC cell line it has been reported that IGF1 downregulates LPL messenger expression and reduces its enzymatic activity [93]. Therefore, IGF1 could play a role in hepatocarcinogenesis by favouring HCV infection. In HuH-7 and HepG2 cell lines, it has been showed that IGF2 downregulation decreases cell proliferation [94]. Additionally, it has been reported that IGF2 gene is a “carrier” for miR-483, an intronic micro-RNA (miRNA), which is able to stimulate cell proliferation in HCC, through the downregulation of its target Socs3 (suppressor of cytokine signaling 3) [95]. IGF2 has also a role in tumour cells migration and angiogenesis. Indeed, in the human HCC cell line HepG2, under conditions of hypoxia, IGF2 messenger expression has been found to be increased, and, in turn, IGF2 has been shown to stimulate VEGF cell production. Moreover, in HepG2, the silencing of IGF2 gene has been shown to reduce the secretion of VEGF in cell supernatants and to decrease the *in vitro* colony formation [96]. In *in vivo* preclinical studies, it has been reported that IGF1 messenger levels are lower in the liver tumour tissue than in the tumour-surrounding tissue or healthy liver tissue of mice harbouring HCC [97]. IGF2 messenger levels are elevated in murine HCCs with enhanced metastatic potential, as compared to murine low invasive HCCs [98]. Dysregulation of IGFs could have a role in the pathogenesis of at least a subset of HCCs. The role of IGF1 is controversial. Several studies have demonstrated that high serum IGF1 and low serum IGFBP3 are associated with an increased risk of prostate, breast, colorectal and lung cancer, and HCC [99-101], probably due to the high bioavailability of mitogenic IGF1. HCC associated with cirrhosis, regardless of HBV and HCV infection, is characterized by significantly lower levels of serum IGF1 than healthy subjects [102,103]. In patients with liver cirrhosis, a condition of IGF1 deficiency is thought to result from the reduced synthetic capability of damaged hepatocytes, as supported by the correlation between IGF1 levels and albumin in cirrhotic patients [104]. In HCC, the reduced binding of GH to the GHR could contribute to the IGF1 deficiency [82]. Additionally, the low circulating levels of IGF1 significantly correlate with advanced clinicopathologic parameters and poor overall survival in patients with HCC [105]. In a study evaluating IGF1 and IGFBP3 levels in 40 cirrhotic patients, 63 HCC patients and 150 healthy subjects, both serum IGF1 and IGFBP3 levels were significantly lower in cirrhotic and HCC patients than in controls. Interestingly, the IGF1/IGFBP3 ratio in HCC patients was significantly higher than in both cirrhotic patients and controls, suggesting that HCC could be associated with an increased IGF1 bioavailability [101]. Therefore, it has been suggested that IGF1/IGFBP3 ratio, more than IGF1 itself, could play a role in hepatocarcinogenesis. Therefore, IGF1 bioactivity could be an attractive parameter [100,106] to be investigated in patients with chronic liver disease and HCC. Conversely to IGF1, serum IGF2 levels in patients with HCC were significantly higher than in patients with cirrhosis and healthy subjects [107-109].

Regarding the expression of IGFs in human liver tissues, it has been reported that IGF1 messenger expression is lower in HCC than in normal liver, while no relevant differences have been found in liver tissues from patients with chronic hepatitis, as compared with normal liver [110,111]. However, IGF1 messenger expression is lower in human chirrotic tissues than in both tumour and normal liver tissues [110,112]. IGF2 messenger has been reported to be overexpressed in human liver with chronic hepatitis, cirrhosis and HCC as compared with normal adult liver [113-116]. Additionally, high IGF2 protein expression has been described in HBV- and HCV-positive HCC tissues, compared with normal and HCC cirrhotic and virus negative tissues [117,118]. The inactivation of adult-specific IGF2 promoter (P1) [119] and the activation of foetal-specific IGF2 promoters (P2-P4) could represent the mechanism underlying IGF2 dysregulation in HCC [120]. Indeed, in human HCC cell lines it has been shown that HBV X protein stimulates P3 promoter activity and HCV core protein is able to increase IGF2 P4 promoter expression [121,122]. Therefore, HBV and HCV infection can promote IGF2 overexpression, which is a common feature of human HCCs. IGF2 transcription is also regulated by aflatoxin B1, an HCC causative toxic compound produced by *Aspergillus* molds, specifically through the activation of P4 promoter. Indeed, aflatoxin B1 induces a mutation in p53 gene, at the level of codon 249 (p53mt249), that strongly increases the activity of P4 [123]. Furthermore, in human HCC tissues it has been demonstrated that VEGF and IGF2 gene-specific single nucleotide polymorphisms are significantly correlated to the expression of metastatic tumour antigen 1 (MTA1), a metastasis-associated protein involved in the increase of cell migration and invasion [124].

In conclusion, IGFs seem to have a role in hepatocarcinogenesis. IGF1 has a main role in influencing HCV infection capability, thus contributing to HCC development. Moreover, the increased IGF1 bioavailability in patients with HCC, probably as a consequence of autocrine secretion by the neoplasm, could be an important factor for tumour progression. IGF2 is highly expressed in human HCC, where it seems to have a stimulatory effect on tumour cell proliferation. Additionally, IGF2 can be responsible, at least partially, of HBV, HCV and aflatoxin carcinogenic effects.

### IGF receptors

The role of IGF receptors, expecially of IGF1R, on the development and progression of HCC has been widely studied.

The expression of IGF1R is significantly increased in HCC in rats [65]. A growing body of evidences suggests an increase in IGF1R expression in human cirrhotic liver, hepatoma cell lines and HCC [64,88,125-128]. In a preclinical setting, an up-regulation of IGF1R expression has been identified in Hep3B, a human hepatoma cell line transfected with p53mt249 [129], and in SNU368, a human HCC cell line expressing HBV protein (HBx) [130]. In physiological conditions*,* IGF1R protein is downregulated by several miRNA [131]. miRNAs are a new class of gene expression regulators that can control cell proliferation and cancer. Particularly, several miRNAs, including miR-122, miR-21, miR-222 and miR-145, seem to play a role in viral-induced liver damage, by regulating hepatocyte infection and proliferation. Among these miRNAs, miR-122 is a liver-specific miRNA abundantly expressed in hepatocytes and known to modulate lipid metabolism, HCV replication and apoptosis [132]. Persistent expression of miR-122 has been detected during hepatic cell differentiation, while miR-122 is barely detectable in primary human HCCs [132,133]. As demonstrated in normal liver cells in physiological conditions, miR-122 has been suggested to suppress IGF1R expression, by binding to the untranslated region of the messenger, which codifies for IGF1R, thus blocking IGF1R translation. This block attenuates IGF1R/Akt signalling, resulting in an increased glycogen synthase kinase-3 beta (GSK-3β) activity, that, in turn, suppresses cyclin D1 expression and cell proliferation. On the other hand, the activated GSK-3β maintains high levels of miR-122 *via* CCAAT/enhancer binding protein alpha (C/EBPα), which enforces IGF1R suppression [134]. In pathological conditions, in particular in response to liver insults by HCV infection, dysregulation of this circuit may result in uncontrolled cell proliferation and, in turn, in hepatocarcinogenesis, due to a reduction of miR-122 expression, phosphorylation of Thr222/226-C/EBPα, an enhancement of IGF1R protein, and phosphorylation of Ser9–GSK-3β [134]. Surprisingly, a preclinical study showed that C/EBPα expression is also upregulated by IGF2; this finding is unexpected considering that the mitogen IGF2 should decrease the expression of tumour suppressor genes such as C/EBPα [135]. Thus, IGF1R play a role in a regulatory circuitry whose dysfunction may contribute to the development of HCC.

In HCC, IGF1R expression seems not to be correlated with tumour size, histological differentiation, capsular invasion and portal venous invasion [136]. The expression of IGF2R gene has been reported significantly reduced in human HCC tissues, compared with surrounding normal liver [137]. IGF2R loss of heterozygosity coupled with intragenic loss-of-function mutations in the remaining allele is a common event in hepatocarcinogenesis [138,139]. Mutations occurring in the IGF2 binding site of IGF2R lead to increased bioavailability of circulating IGF2, thus allowing IGF2 to activate IGF1R and IR, and to enhance cell proliferation. This molecular event may favour HCC progression [140]. Figure 3 shows the intracellular signalling induced by IGF1R and IGF2R.

In conclusion, IGF1R is overexpressed in conditions predisposing to HCC, such as cirrhosis, as well as in HCC. IGF1R overexpression facilitates IGF2 oncogenic activity. The reduction of miR-122 expression, potentially induced by HCV infection, is an important regulatory mechanism of IGF1R overexpression, representing a relevant link between IGF pathway and viral agents, in particular HCV, and potentially between IGF pathway and development of HCV-induced HCC.

### GHBP

A decrease of circulating GHBP levels has been described in patients with cirrhosis [141,142]. An increase in GHBP levels have been reported in patients with non-alcoholic fatty liver disease (NAFLD), as compared to healthy subjects [143]. Moreover, it has been clearly demonstrated that the most severe cirrhosis have significantly decreased circulating GHBP levels [141]. A hypothesis for the increased GHBP levels in NAFLD and the reduced GHBP levels in different chronic liver diseases, including cirrhosis, might be that the two isoforms of the GHR have a different pattern of expression in different liver diseases. In fact, it has been demonstrated that a truncated form of the GHR is normally expressed at low levels compared with the full-length receptor, but shows a higher ability to generate GHBP [144-146]. Some authors demonstrated that in cirrhotic liver, the expression of the truncated form of GHR was reduced compared with the full-length isoform, and this may contribute to the lower GHBP levels found in patients wirh cirrhosis [147]. Conversely, in NAFLD, some authors have hypothesised a higher expression of the truncated form of the GHR and a higher GHBP production [143]. Another hypothesis for the increased GHBP levels in NAFLD may be a higher production rate of GHBP from adipose tissue; indeed, the abdominal fat, which is increased in patients with NAFLD, is correlated with GHBP levels [148].

To our knowledge, neither in vitro nor in vivo studies investigating the role of the GHBP in hepatocarcinogenesis have been conducted up to date.

### IGFBPs

IGFBPs have high affinities for IGFs, thereby the activities of either IGF1 or IGF2 are modulated by their association with IGFBPs. This evidence suggests that IGFBPs exert a protective effect towards the IGF-induced cell proliferation, through the restriction of the availability of these ligands for binding to IGF1R. IGFBPs regulate proliferation, differentiation and apoptosis of various cell types in an IGF-dependent and -independent manner [48,149]. IGFBP3 is the most commonly investigated binding protein. In preclinical setting, IGFBP3 has been demonstrated to induce a significant reduction of cell proliferation and invasion, in several human HCC cell lines (HAK-1B, KIM-1, KYN-2 and HepG2), through the reduction of the bioavailability of endogenous IGF2 for cell surface receptor binding [150,151]. Moreover, IGFBP3 attenuates also the proliferative action of IGF1. Indeed, in HepG2 cell line, it has been demonstrated that, in the presence and absence of IGF1, IGFBP3 attenuated the IGF1-induced proliferation at low concentrations and completely abolished the IGF1-induced proliferation at high concentrations. These results suggest that, at least in HepG2, IGFBP3 attenuates IGF1-induced proliferation by binding IGF1 and, therefore, reducing IGF1 bioavailability to its receptor [151]. Immunohistochemical analysis of HCC tissues, which express IGFBP3, reveals abnormalities in TGF-β and/or retinoblastoma protein (Rb) pathways. These results opened the question whether IGFBP3 may mediate growth suppression *via* the TGF-β and/or Rb pathways in HCC; however, this issue needs to be further investigated [152].

The expression of IGFBP1, 2, 3 and 4 in cirrhotic liver is similar to normal liver tissues, but it is significantly downregulated in HCC tissues, compared with normal and cirrhotic liver [153]. In HCC patients, reduced expression of IGFBP3 has been found to be significantly correlated with tumour size, histological differentiation, capsular invasion, portal venous invasion and poor survival [150]. Promoter hypermethylation of IGFBP3 gene has been suggested as a potential mechanism for IGFBP3 downregulation in HCC [154]. IGFBP3 levels were negatively correlated with liver function measured as Child-Pugh class in patients with liver cirrhosis and, weaklier, in those with HCC, as compared with healthy subjects [101,107]. The estimation of serum IGF1, IGF2 and IGFBP3, together with Child-Pugh score, is more effective in predicting liver dysfunction and its severity, compared to Child-Pugh score alone [107].

In conclusion, IGFBP3 is downregulated in HCC and it is correlated with important clinical parameters. Therefore, IGFBP3 could play an indirect role in HCC development by reducing IGF1 bioavailability to its receptor, and could be a molecular target for novel therapeutic strategies in HCC patients.

### Somatostatin and somatostatin receptors

An *in vitro* study provided the evidence that HepG2 cell line does not produce SST but it does produce cortistatin (CST), a neuropeptide showing high structural homology with SST and binding to all SSTRs [155]. SSTRs expression has been heterogeneously demonstrated by *in vitro* studies in human HCC cell lines, including HepG2, HepB3, HuH-7, SMMC-7721 and Bel7402, with different findings among the various cell lines, as well as in the same cell line investigated in different studies [27,70,155-158]. However, SSTR2 expression has been homogeneously documented in the entire series of cell lines [27,70,155-158]. Immunocytochemistry analysis showed that SSTRs were located mainly intracellularly in HepG2 and HuH-7 cells [155,158]. It has been suggested that endogenous production of CST may be responsible for SSTRs internalisation and modification in these cell lines [155].

The expression of SSTR2 has been demonstrated in tissues of mice in which HCC was induced by treatment with diethylnitrosamine [159]. The expression of SSTRs has been largely demonstrated in both resectable and unresectable human HCC tissues [27,160-163]. SSTRs protein expression has been reported to be unrelated to tumour stage, differentiation, histological tumour type and/or underlying liver disease [164]. However, a recent study evaluating the expression of SSTR2 and SSTR5 in 76 tumour samples from patients with HBV-related operable HCC, found that, in this particular subset of patients, the mean survival time was longer in the subgroup of patients expressing high SSTR2 and SSTR5, and that, at multivariate Cox analysis, tumour expression levels of SSTR2 were an independent prognostic marker [161]. Moreover, progressive upregulation of SSTRs during the different stages of hepatocarcinogenesis has been also documented [27]. In this study, SSTRs were not expressed in hepatocytes from normal liver, whereas they were expressed in cirrhotic liver and HCC, although with variable intensity [27]. Somatostatin receptor scintigraphy with ^111^indium pentetreotide (Octreoscan) has been used to screen HCC patient for SSTR2 and/or SSTR5 positivity. In two different cohort of patients including 127 and 70 patients, SSTR2 positivity was registered in 48% and 35.7% of patients, respectively [165]. These data demonstrated that at least a subgroup of patients with HCC presents SSTR2 and/or SSTR5 expression. In HCC, the positivity at Octreoscan was not related with the main clinical parameters [165,166]. However, the role of SSTR expression in HCC tissues and of Octreoscan positivity in patients with HCC should be better addressed.

In conclusion, SSTRs are expressed in HCC cell lines as well as in cirrhotic tissues and HCC, but SSTRs expresssion seems to be not correlated with tumour stage, grading and prognosis.

### Targeting GH-IGF-SST system in hepatocellular carcinoma

**Figure 5:**
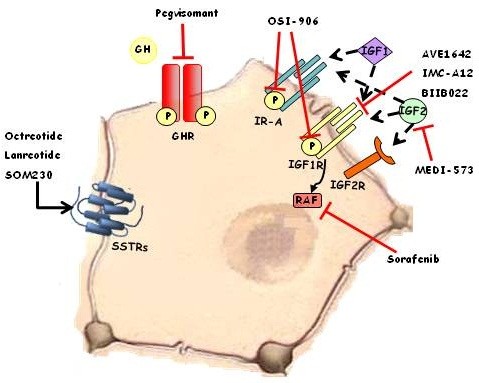
**Several strategies in the therapeutic considerations involving GH-IGF-SST system in the treatment of HCC.** Dotted black arrows: activation by endogenous ligand. Black arrow: activation by exogenous ligand. Red lines: blocking of ligands and receptors.

Several approaches to GH-IGF-SST system targeting have been used as novel therapeutic strategies in HCC, and some others are currently under evaluation [167,168]. Generally, molecular therapeutic strategies include the use of antibodies, which can have anti-ligand and/or anti-receptor activity, the use of small molecules inhibitors, which can interfere with key enzymatic functions, and the use of synthetic receptor agonists or antagonists [25,169,170]. All these approaches can interfere with cell proliferation and/or, specifically, with apoptosis. Monoclonal antibodies and small molecule inhibitors can be used to target receptors, particularly growth factor-RTK. These growth factor-RTK targeting approaches can be combined with different small molecule inhibitors targeting cytoplasmic oncogenic kinases [170]. Figure 5 shows the therapeutic strategy involving GH-IGF-SST system in the treatment of HCC.

### GH and GH receptor

The modified GH analogue pegvisomant was the first specific GHR antagonist to be genetically engineered and produced by the pegylation of mutant GH [171]. The pegylation has the benefit to increase the half life of drugs and sterically interfere with GHR dimerization at the cell surface, which is essential for GHR activity [171]. The anti-tumour activity of pegvisomant has been tested in breast cancer, colorectal cancer and meningiomas [172-174]. In *in vivo* studies on animals, pegvisomant caused tumour shrinkage in nude mice xenografted with a human breast cancer cell line (MCF-7) and with two different human colorectal cancer cell lines (COLO 205 and HT-29); in these models a decrease of cell proliferation and the induction of apoptosis were clearly demonstrated [172,173]. Pegvisomant has been also demonstrated to significantly reduce the growth of meningioma in athymic mice xenografted with human meningioma primary cultures [174].

To the best of our knowledge, no studies have evaluated the effects of pegvisomant in preclinical or clinical models of HCC, but the GHR expression and GH effect on HCC permit to hypothesize a role of pegvisomant in this tumour.

### IGF pathway

IGF pathway has been a target for the treatment of various tumours for long time. Two kinds of target therapy are usually used in clinical setting: anti-ligand and anti-receptor drugs treatment. A list of the currently available agents targeting IGF pathway, evaluated in clinical trials for the treatment of HCC, is shown in Table 1.

**Table 1:** Currently available agents against the IGF pathway evaluated in clinical trials for the treatment of HCC

**Compound**	**Company**	**Mechanism of action**	**Phase of clinical development**	**Trial Status**	**Intervention**	**Type of Cancer**
***MEDI-573***	*MedImmune LLC*	*Fully Human mAb anti- IGF1 and –IGF2*	*1b/2*	*Completed*	*In combination with sorafenib*	*Unresectable or metastatic HCC*
***IMC-A12***	*National Cancer Institute*	*Fully Recombinant Human mAb anti- IGF1R*	*2*	*Completed*	*Alone*	*Adult Primary, advanced, localized unresectable, recurrent HCC*
	*National Cancer Institute*		*1*	*Active, not recruiting*	*In combination with sorafenib tosylate*	*Advanced HCC*
	*Eli Lilly and Company*		*2*	*Active, not recruiting*	*In combination with sorafenib*	*Advanced HCC*
***BIIB-022***	*BiogenIdec*	*Human IgG4P nonglycosylated antibody anti-IGF1R*	*1b*	*Completed*	*In combination with sorafenib*	*Advanced HCC*
***AVE-1640***	*Sanofi-Aventis*	*Humanized mAb anti- IGF1R*	*1/2*	*Completed*	*Alone and in combination with sorafenib and erlotinib*	*HCC not eligible for local treatment*
***OSI-906***	*AstellasPharmaInc*	*Small molecule inhibitor of IGF1R*	*2*	*Completed*	*Alone*	*With advanced HCC after failure of first-line treatment with sorafenib*

Data from clinicaltrials.gov.

#### Anti-ligand approach

MEDI-573, a human IgG2 monoclonal antibody (mAb), is one of the first monoclonal antibodies produced against ligands. This antibody neutralises both IGF1 and IGF2 without cross-reactivity to insulin. However, since it acts by neutralising IGFs, it blocks the IGF-induced activation of both IGF1R and IR downstream signalling pathways. This double effect has been confirmed by *in vivo* experiments in athymic mice xenografted with two mouse fibroblast cell lines: P12, engineered to overexpress human IGF1 and IGF1R proteins but not human IGF2, and C32, engineered to overexpress IGF2 and IGF1R proteins but not IGF1 [175]. Moreover, in this study, it has been demonstrated that MEDI-573 significantly inhibited the growth of P12 and C32 tumour outgrowths in xenografted nude mice [175]. To date, MEDI-573 has been tested in a Phase1 study (NCT00816361) in subjects with advanced solid tumours. In a currently completed Phase1b/2 study (NCT01498952), an open-label, randomized study, MEDI-573 has been tested in combination with sorafenib, a small molecule inhibitor of several tyrosine protein kinases (VEGFR and PDGFR) and Raf kinases, in adult subjects with unresectable or metastatic HCC; however, the results of this study are not currently available.

Since IGFPB3 naturally binds the ligands of the IGF pathway, non-glycosylated human recombinant IGFBP3 (rhIGFBP3) has been proposed as ligand antagonist. In *in vitro* studies, rhIGFBP3 has been reported to significantly inhibit cell proliferation of murine lung metastatic (M-3LL) and human colon metastatic (LoVo) cell lines [176]. Treatment of HepG2 cells with human rhIGFBP3 led to a significant reduction in cell proliferation and attenuated the mitogenic activity of IGF1 [151].

#### Anti-receptor approach

IGF1R is considered the main receptor responsible for the mitogenic effects of the IGF axis [43], therefore, it represents an attractive target for anti-cancer therapy. Drugs targeting the IGF1R are also called IGF1R-blockers and include: anti IGF1R monoclonal antibodies such as CP-751,871, AVE1642/EM164, IMC-A12, SCH-717454, BIIB022, AMG 479 and MK-0646/h7C10, and small molecules RTK inhibitors such as OSI-906 [43]. Several in vitro and in vivo studies have evaluated the effects of these compounds in different types of preclinical cancer models, as recently reviewed [43,177]. This review will focus on the studies evaluating the effects of IGF1R-blockers in HCC.

IMC-A12, also known as cixutumumab, is a fully human monoclonal IgG1 antibody that binds IGF1R with high affinity, inhibits ligand-dependent receptor activation and downstream signalling, and also mediates IGF1R internalization and degradation [178]. IMC-A12 has shown antitumoral activity against a wide range of human tumour types in in vitro studies. The effect of IMC-A12 has been evaluated in human breast (MCF7), pancreas (BxPC-3), and colon (Colo205) carcinoma cell lines, in which the antibody inhibits cell proliferation and induces cell apoptosis [179]. In the same study, IMC-A12 has also shown activity against a human tumour in both in vivo xenograft and orthotopic models. Immunohistochemical studies on tumour biopsies from mice bearing MCF7 tumours, treated with IMC-A12, showed a 20% reduction of proliferating cells and an increase of apoptotic cells [179]. IMC-A12 treatment induced significant antitumour activity also in Colo205 and BxPC-3 xenografts, affecting tumour growth and showing >70% and 80% of growth inhibition, in Colo205 and BxPC-3 xenografts, respectively [179]. IMC-A12 has also shown potent activity, as a single agent, against xenograft models of human non-small cell and small cell lung carcinoma, as well as in models of prostate, renal, thyroid and head and neck carcinoma, multiple myeloma and sarcoma [180-183]. Nowadays, a Phase 2 study (NCT00639509) in patients with primary, advanced, localized unresectable, recurrent HCC, has been completed. The results of this study showed that IMC-A12 did not have the expected effects in this cohort of unselected HCCs. No correlation was found between IGF1R positive staining and treatment outcome [184]. Phase 1 (NCT01008566) and Phase 2 (NCT00906373) trials with IMC-A12, in combination with sorafenib, are ongoing and the results of these studies are still awaited.

AVE1642 is a humanized version of the murine IGF1R mAb EM164. In in vitro studies in human HCC cell lines (HepG2, Hep3B, HuH-7, HuH-6, PLC/PRF5), it has been demonstrated that AVE1642 inhibits cell proliferation by preventing the activation of signalling in response to exogenous IGF1 and IGF2, but not insulin, supporting the IGF1R specifity of this antibody [88]. In the same study, it has been demonstrated that AVE1642 is able to downregulate Akt phosphorylation, and this effect was increased when AVE1642 was combined with gefitinib (EGFR inhibitor) or rapamycin (mTOR inhibitor) [88], supporting the efficacy of combined treatments in HCC. AVE1642 has been tested in a completed Phase 1/2 study (NCT00791544, as single agent and in combination with other anti-cancer therapies, in patients with advanced HCC not eligible for local treatment; the results of this study are still awaited.

OSI-906 is a potent and selective small molecule RTK inhibitor, targeting both IGF1R and IR. This drug compared with the other class of IGF1R blockers has the advantage to inhibit also the IGF2-induced IRA activation, which has been reported to favour growth in cancer [40]. OSI-906 potently inhibits ligand-dependent auto-phosphorilation of both IGF1R and IR, preventing the activation of pAkt, pERK1/2, and pp70S6k, and thus inhibiting cell proliferation [185]. OSI-906 displays in vitro antiproliferative effects in several human tumour cell lines and robust in vivo anti-tumour effects in IGF1R-dependent mice xenograft model of fibrosarcoma [185]. In an in vitro study, OSI-906 showed inhibitory effects on cell proliferation in several HCC cell lines, expecially in those displaying epithelial phenotype. The inhibitory effect of OSI-906 results in the inhibition of the IRS/Akt pathway [186]. Recently, a randomized, placebo-controlled, double-blind Phase 2 study (NCT01101906) has been concluded on patients with advanced HCC after failure of first-line treatment with sorafenib, but the results of this study are still awaited. Another recent Phase 2 study (NCT01334710) performed in patients with advanced HCC receiving OSI-906 in combination with sorafenib has been suspended, for safety reasons, by the pharmaceutical company. BIIB022, a human anti-IGF1R mAb, has been used in a Phase 1/b study (NCT00956436) in combination with sorafenib in patients with advanced HCC, but the results of this study are still awaited.

IGF1R-blockers have been generally documented to be well tolerated, but extended blockade of IGF1R signalling could potentially produce clinical signs and symptoms similar to those of severe untreated growth hormone deficiency, including visceral adiposity, dyslipidemia, deterioration of cardiac performance, osteoporosis, and impairment of physical and psychological performance [187,188]. Moreover, several metabolic side effects such as insulin resistance and gluconeogenesis, could result secondary to increased GH levels in the absence of IGF1R function [43].

In conclusion, preclinical studies suggest that agents targeting IGF axis could provide a promising alternative treatment in HCC patients. However, the results of clinical trials evaluating the effects of drugs targeting this axis in HCC patients are awaited.

### Somatostatin receptor agonists (SA)

SSTRs are a treatment target in some types of tumours. Given the short half-life of native SST, several synthetic SA have been developed and are currently used in clinical practice, mainly to treat patients with neuroendocrine tumours (NET) [25]. Among these, the most important are: octreotide, which binds with high affinity to SSTR2 and with reduced affinity to SSTR3 and SSTR5; lanreotide, which primarily binds with high affinity to SSTR2 but shows reduced or no binding to SSTR1, 3, 4 and 5 subtypes; pasireotide, which has high affinity for SSTR5 but it also binds SSTR2, SSTR3 and SSTR1, with decrescent affinity [25,189]. Several *in vitro* studies using cell lines transfected with SSTRs indicate that all receptor subtypes (SSTR1-5) may mediate the inhibition of cell proliferation, whereas specific receptor subtypes (SSTR2, SSTR3) may mediate the induction of apoptosis [190]. These effects are regulated primarily *via* MAPK pathway and through the activation of phosphotyrosine phosphatases [191,192]. The growth inhibition effects of SA might be also induced by the restoration of functional gap junctions [192,193]. Moreover, SA could upregulate the tumour suppressor protein p53 and activate the pro-apoptotic member of the Bcl-2 protein family, Bax, thus triggering apoptosis [193].

In a subset of patients with NET, SA have been found to improve clinical syndrome, to control hormonal secretion and to inhibit tumour growth [26,194,195]. Additionally, radiolabeled-SA have been developed and are clinically used to visualize NETs or to perform radiometabolic treatments in NET patients [25]. SA, alone or in combination with other antitumour treatments, have been associated with some favourable clinical outcomes in not classical neuroendocrine solid tumours, such as prostate cancer, that has been found to express different SSTR subtypes, including SSTR2 and/or SSTR5 [26]. Therefore, it has been suggested that SSTRs might be useful also in the clinical management of patients with other types of tumours expressing SSTRs [25].

Many studies have tried to address the role of SA in HCC treatment but, up to date, both preclinical and clinical data are still controversial.

The effects of SA in both normal and tumour hepatic cells have been investigated in several *in vitro* studies. The activation of SSTRs results in the inhibition of proliferation in normal liver cell lines and in some, but not all, neoplastic liver cell lines [27,70,155-157,196]. Particularly, in the normal liver cell line L-02, octreotide was found to inhibit cell proliferation and, at the highest dose, it was found to induce apoptosis [70,189]. The same study also demonstrated that octreotide was able to inhibit cell proliferation and to induce apoptosis in HepG2 and SMMC-7721 HCC cell lines, which appeared to be more sensitive to the proapoptotic effects of octreotide than normal liver cell line L-02 [70]. However, these results must be considered carefully, since the drug concentration used in these *in vitro* experiments was higher than the maximal dose generally achieved in the therapeutic regimen of the SA, in the clinical practice [70]. The effects of octreotide on cell proliferation have been also tested in human cell lines with high (MHCC97-H) and low (MHCC97-L) metastatic potential. These cells were insensitive to the treatment with octreotide, consistently with their lack of SSTRs expression [197]. None of several subtype specific SA [L-797,591 (SSTR1 agonist), L-779,976 (SSTR2 agonist), L-796,778 (SSTR3 agonist), L-803,087 (SSTR4 agonist), and L-817,818 (SSTR5 agonist)] affected proliferation or apoptosis in two human HCC cell lines (HepG2 and HuH-7), but L-797,591 inhibited the migration of HepG2 and HuH-7 cells in the presence of chemotactic stimuli [27]. In HepG2 and HepB3 HCC cell lines, AN-238, which is a cytotoxic agent consisting of 2-pyrrolino-doxorubicin (AN-201) conjugate to a well-characterized somatostatin octapeptide carrier, RC-121 (binding SSTR2 and 5) [198,199], was able to inhibit cell proliferation by inducing cell cycle block in sub-G1 phase and to induce apoptosis by triggering DNA fragmentation and cleavage of poly ADP-ribose polymerase (PARP) protein [157]. The role of the new universal SA pasireotide on HCC cell proliferation has also been recently investigated. In HepG2 cell line, treatment with pasireotide, alone or combined with celecoxib, inhibited cell viability in a dose dependent manner [200].

SA showed some antitumoral effects also in *in vivo* preclinical models of HCC. Treatment with octreotide inhibited tumour growth in nude mice bearing HCC xenografts [148]. Additionally, octreotide and pasireotide induced tumour necrosis, probably inhibiting VEGF expression [197,201,202]. The anti-vascular effects of SA have been supported also by *in vitro* and *in vivo* studies in experimental models of angiogenesis [203-205]. In an animal model of hepatocarcinogenesis, the effects of lanreotide on HCC prevention have been evaluated. In this study rats were treated chronically with a carcinogenic drug and they were assigned to three treatment groups: rats receiving lanreotide from the beginning of the experiment, rats receiving lanreotide at the onset of fibrosis, and rats not receiving lanreotide (control group). In both the groups receiving lanreotide the frequency of HCC was decreased of about 60%, compared with control group. Decrease in hepatocytes proliferation and inhibition of fibrosis were also demonstrated. Additionally, when given at the start of the experiment, lanreotide dramatically decreased the levels of angiogenic factors and enhanced apoptosis [206].

Several case reports and clinical trials regarding the use of SA in the management of patients with HCC have been reported. In one of the first case reports on advanced HCC, lanreotide slow-release (lanreotide SR) was administered in order to manage para-neoplastic diarrhea. After two intramuscular injections of lanreotide SR at a dose of 30 mg every 10 days, the alpha-fetoprotein (AFP), an important tumour marker for HCC, levels were drastically reduced and when lanreotide therapy was continued, given the good tolerance to the treatment, a slight decrease of tumour size with pronounced signs of necrotic changes were also registered [207]. The long-acting release formulation of octreotide (octreotide LAR), at a dose of 10 mg administered monthly, has been proven to be useful in the treatment of HCC, resulting in AFP levels normalization and a complete and prolonged regression of the tumour in a patient with not resectable HCC [208]. The clinical use of octreotide LAR has been shown to be useful in a patient with metastatic HCC, in which it improved quality of life (QoL) and reduced AFP levels and tumour size [209]. Recently, lanreotide SR at the dose of 30 mg, administered twice a month, has been used to treat a patient with recurrent HCC and lung and mediastinal nodes metastases, after primary tumour complete resection. In this patient, the expression of SSTR2 was demonstrated on both primary tumour and metastases, and three months of treatment with lanreotide SR 30 mg administered twice a month induced a decrease in the size of the mediastinal nodes and complete disappearance of the lung nodes [210]. Schematic descriptions of case reports are presented in Table 2.

**Table 2:** Case reports of HCC treatment with SA monotherapy

**Publication**	**Type of study**	**Number of enrolled patients and controls**		**Type of Cancer**	**SA used for treatment**	**Response to treatment-outcome**
		***Patients***	***Controls***			
Raderer 1999 [207]	Case report	1	-	Advanced HCC	LAN SR (30 mg/10d)	↓AFP, ↑TN, SD
Siveke 2003 [208]	Case report	1	-	Advanced HCC	OCT LAR (10 mg/28d)	CR, ↓AFP
Deming 2005 [209]	Case report	1	-	Metastatic HCC	OCT LAR (30 mg)	↑QoL, ↓AFP, PR
Borbath 2012 [210]	Case report	1	-	Metastatic HCC with lung and mediastinal nodes, HBV+,	LAN SR (30 mg/14d)	PR

*CR*, Complete response.

*PR*, Partial response.

*QoL*, Quality of life.

*SD*, Stable disease.

*TN*, Tumor necrosis.

*AFP,* Alpha fetoprotein.

Despite these positive case reports, to date several randomized and non-randomized clinical trials conducted in unresectable HCC patients using octreotide or lanreotide reported conflicting results. Several prospective non-randomized trials investigated the role of octreotide LAR or lanreotide, as summarized in Table 3. Dimitroulopoulos *et al*. enrolled 28 cirrhotic patients with advanced HCC. Octreoscan for the detection of SSTRs was performed in all cases. In patients showing intense uptake in the liver, octreotide was administered as follows: all patients started treatment with octreotide 0.5 mg, administered subcoutaneously every 8 hrs for 6 weeks. After 4 weeks, treatment with ocreotide LAR 20 mg/4 weeks was added, and between week 4 and week 6 both treatments were administered. From week 8, patients received only octreotide LAR 20 mg every 4 weeks, uptitrated to 30 mg every 4 weeks from week 12. Thirteen patients unable to receive treatment were used as control group. Neither AFP reduction nor decrease of tumour mass were observed, but treatment improved both median survival time and QoL [211]. Gill *et al*. compared the outcomes of 22 patients with inoperable HCC treated with octreotide and 20 HCC patients that refused treatment due to socio-economic issues (control group). Patients received octreotide 100 μg subcutaneously, twice a day for 2 weeks. This treatment was followed by a monthly intramuscular administration of 20 mg octreotide LAR. In this study octreotide LAR treatment induced tumour size regression, AFP level decrease and QoL improvement [212]. In the non-randomized study of Samonakis *et al*. the survival of 32 patients with inoperable HCC treated with long acting SA (octreotide LAR or lanreotide SR) has been compared with a historical control group of 27 untreated patients. In these patients, the use of long acting SA appeared to have a positive effect on survival and QoL in inoperable HCC cases [213]. Plentz *et al*. recruited 41 patients with advanced HCC and cirrhosis, including predominantly alcoholic, and HBV and HCV-induced secondarily cirrhosis, treated with short-acting and long-acting octreotide. Patients started the treatment with 50 mg of subcutaneously administrated short-acting octreotide, 3 times a day in the first week. The octreotide dose was increased by 50 mg per application each week until a final dose of 250 mg octreotide, 3 times a day, was reached. After this regimen, patients received 30 mg intramuscular octreotide LAR, once every 4 weeks. These patients were compared with a group of patients treated with transarterial chemoembolization (TACE) and no difference in median survival was registered [214]. Schoniger-Hekele *et al.* studied retrospectively the influence of octreotide LAR monotherapy on survival of patients with HCC and compared it to BCLC stage-matched patients who received either TACE, multimodal therapy, or palliative care only. Their study demonstrated that in the subgroup of 55 patients, classified as BCLC stage B, survival rates of patients with "active" treatments (octreotide LAR, TACE or multimodal therapy) were significantly higher than those of patients who received only palliative care. In both BCLC stage A and B patients, octreotide monotherapy showed a similar outcome, compared with patients who received TACE or multimodal therapy [215]. Slijkhuis*et al*. investigated the effect of octreotide LAR in a prospective uncontrolled study. Initially, 30 patients received short-acting octreotide to ensure drug tolerability. Thereafter, 24 patients received octreotide LAR 30 mg every 4 to 6 weeks. In this study, median time to tumour progression was 3.6 months, and median survival was 5.1 months; 7 patients (29%) had stable disease (median duration of 8.0 months) with 2 patients demonstrating disease stability for 24 months [216]. In a study of our research group, 20 patients with advanced HCC were treated with octreotide LAR 30 mg/month and a pool of 40 patients with HCC with tumour staging and liver function comparable to the study patients was retrospectively selected as control. The patients treated with octreotide LAR showed a significantly higher mean survival rate, compared with controls, and patients’ survival was significantly correlated with SSTR2 messenger expression in the tumour [217]. In a phase 2 multicenter study (So.LAR.), 55 patients with advanced HCC, Child-Pugh A or B, received sorafenib at a dosage of 800 mg/day for 28 days with a following week of rest and octreotide LAR at a dose of 40 mg, administered every 28 days. Treatment was well tollerated and partial response was documented in 10%, whereas a stable disease was achieved in 66% of patients after treatment. This study demonstrated that this combination of treatments can be a safe and effective option [218]. In a following study the authors investigated, in patients included in the So.LAR study, whether oxidative stress evaluated in biological samples, including both serum and peripheral blood mononuclear cells (PBMC), and pERK activation status in PBMC, could be predictive of response. The results of this study suggested that the levels of nitric oxide activity were correlated with the prognosis of HCC patients treated with So.LAR schedule and that the determination of both pERK expression in PBMC and the oxidative stress status could have a value in the prediction of response to sorafenib *plus* octreotide therapy in HCC patients [219].

**Table 3:** Non randomized clinical trials of HCC treatment with SA monotherapy

**Publication**	**Type of study**	**Number of enrolled patients and controls**		**Type of Cancer**	**SA used for treatment**	**Response to treatment-outcome**
		***Patients***	***Controls***			
Dimitroulopoulos 2002 [211]	NRCT	15*	13^#^	Advanced HCC	OCT LAR (20 to 30 mg/28d)	↑S, ↑QoL
Gill 2005 [212]	NRCT	22*	20^#^	Advanced HCC	OCT LAR (20 mg/28d)	↑S, ↑QoL, ↓AFP, PR
Samonakis 2002 [213]	NRCT	32*	27^#^	Advanced HCC	NS	↑S, ↑QoL,
Plentz 2005 [214]	NRCT	41*	33▪	Advanced HCC	OCT LAR (30 mg/28d)	=S
Schoniger-Hekele 2009 [215]	NRCT	25*	39†	Advanced HCC	OCT LAR (30 mg/28d)	↑S
		or 17▪				
		or 17▫				
Slijkhuis 2005 [216]	NRCT	30*	-	Advanced HCC	OCT LAR (30 mg/28d)	SD

*NRCT*, Non randomized clinical trial.

*AFP,* Alpha fetoprotein.

*S*, Survival.

*TACE,* Transarterial chemoembolization.

*PR,* Partial response.

*SD*, Stable disease.

*NS,* Not specified.

*QoL*, Quality of life.

*: treated patients.

*#*: untreated patients.

*▪:* TACE treatment.

▫: multimodal therapy.

†: palliative.

To our knowledge, five randomized clinical trials have been performed to investigate the role of octreotide in the management of unresectable HCC, as presented in Table 4. Kouroumalis *et al.* enrolled 58 patients and randomly assigned them to two groups: 28 patients received octreotide 250 μg twice a day, whereas 30 patients did not receive any treatment and were considered as control group. Octreotide administration significantly improved median survival and cumulative survival rate at 6 and 12 months, as compared with control group, by decreasing AFP levels and by improving QoL after 6 months of treatment [220]. Yuen *et al*. performed a study with 70 patients, randomized in octreotide LAR treated group and placebo group and did not find a significant improvement in survival rate, AFP levels or QoL [221]. The value of scintigraphic uptake for predicting the therapeutic response to SA has been proven for NET [222]. Dimitroulopoulos *et al*. recruited 127 patients: 61 positive and 66 negative to Octreoscan. Patients positive to Octreoscan were randomized in two groups: 31 patients were treated with octreotide administered subcutaneously at the dose of 0.5 mg every 8 hrs for 6 weeks and then octreotide LAR at the dose of 20 mg at the end of week 4–8; the remaining 30 patients received placebo. A significantly higher survival time and QoL were observed in the octreotide treated group, as compared with the control group and to the SSTRs negative group [166]. Becker *et al*. conducted a randomized, controlled trial, analyzing 119 patients, divided in 60 patients treated with octreotide LAR at the dose of 30 mg monthly, and 59 patients treated with placebo. They observed no survival improvement and no AFP reduction in HCC patients treated with octreotide LAR, compared with patients treated with placebo [223]. Barbere *et al*. enrolled 272 patients randomly assigned to octreotide LAR treated group (135) and to placebo group (137). The results of this study established that octreotide does not prolong patients’ survival and has a negative impact on QoL [224]. In summary, among the randomized trials, only the two Greek studies reported benefits from octreotide treatment, whereas the German, French and Chinese trials did not show any advantage from the therapy with octreotide. Because of the contradictory results of all these studies, the use of SA in the management of HCC is still matter of debate. These confliting results could depend on the heterogeneous methodology used and heterogeneous population enrolled in the study. Indeed, both the positive studies had a high proportion of HCV related HCC and a low proportion of alcoholic related HCC. Additionally, only one of the positive studies explored the effects of octreotide in a cohort of patients selected on the basis of octreoscan positivity [166]. Overall, the results of these studies demostrated that approximately 40% of HCC patients responded to SA treatment with an improvement of survival rate and an improvement of QoL, but some subsets of patients, such as the Octreoscan-positive or those with HCV-related HCC, might be better candidates for this treatment. However, further studies are mandatory to better address the role of SA in the management of patients with HCC and to better explore the role of aetiology, SSTR expression, or Octreoscan positivity, as predictors of response to this treatment. Lastly, to our knowledge, no data are available regarding the use of pasireotide in patients with HCC.

**Table 4:** Randomized clinical trials of HCC treatment with SA monotherapy

**Publication**	**Type of study**	**Number of enrolled patients and controls**		**Type of Cancer**	**SA used for treatment**	**Response to treatment-outcome**
		***Patients***	***Controls***			
Kourumalis 1998 [220]	RCT	28*	30^#^	Advanced HCC	OCT SC (500 μg/d)	↓AFP,↑S, ↑QoL
Yuen 2002 [221]	RCT	35*	35°	Advanced HCC	OCT LAR (30 mg/28d)	None
Dimitroulopoulos 2007 [166]	RCT	24* (Octreoscan +)	30° (Octreoscan +)	Advanced HCC	OCT LAR (20 to 30 mg/28d)	↑S, ↑QoL
			66^#^ (Octreoscan -)			
Becker 2007 [223]	RCT	60*	59°	Advanced HCC	OCT LAR (30 mg/28d)	None
Barbare 2009 [224]	RCT	135*	137°	Advanced HCC	OCT LAR (30 mg/28d)	=S, ↓QoL

*RCT,* Randomized clinical trial.

*AFP,* Alpha fetoprotein.

*S*, Survival.

*QoL*, Quality of life.

*: treated patients.

*#*: untreated patients.

*°*: placebo controls.

In conclusion, both preclinical and clinical data suggest that SA might have antitumoural effects in a subset of HCC, further studies are required to better define the role of SA in the management of HCC patients.

## Conclusions

GH-IGF-SST system seems to play a role in the development and progression of HCC, although the real impact of this system, either in physiologic or pathologic conditions, on hepatocarcinogenesis is still far from being completely understood. GH-IGF-SST system might potentially represent a target for treatment of HCC. However, drugs targeting IGF pathway and SSTRs seem to be less promising than expected, although clinical trials in selected cohorts of patients or with combined treatment could give better results. Therefore, an open challenge in this field is to define whether GH-IGF-SST system is a good target for treatment in specific subgroups of HCC patients, whether there are predictive biomarkers that can help to early identify the patients potentially responsive to this treatment, and whether combined therapy with multiple drugs targeting this pathway can be more effective than drugs used as monotherapy.

## Abbreviations

AFP: alpha-fetoprotein; AKT/PKB: protein kinase B; ALS: acid-labile subunit protein; CSC: cancer stem cells; CST: cortistatin; DOX: doxorubicin; EC: endothelial cell; EGFR: epidermal growth factor receptor; ERK: extracellular signal-regulated kinases; GC: glucocorticoid; GH: growth hormone; GHBP: growth-hormone binding protein; GHR: growth hormone receptor; GHRH: growth-hormone-releasing hormone; GR: glucocorticoidreceptors; HCC: hepatocellular carcinoma; HSC: hepatic stellate cell; HSPG: heparansulfate proteoglycans; IGF1: insulin-like growth factor 1; IGF1R: insulin-like growth factor 1 receptor; IGF2: insulin-like growth factor 2; IGF2R: insulin-like growth factor 2 receptor; IGFBP: insulin-like growth factor binding protein; INS: insulin; IR: insulin receptor; IRS: insulin receptor substrate; JAK: janus kinase protein; KC: Kupffer cell; LAR: long-acting release; LDL: low-density lipoprotein; LPL: lipoprotein lipase; LVPs: lipoviroparticles; MAPK: mitogen-activated protein kinase; MTA1: metastatic; mTOR: mammalian target of Rapamycin; NAFLD: non-alcoholic fatty liver disease; NET: neuroendocrine tumours; PARP: poly ADP-ribose polymerase; PBMC: peripheral blood mononuclear cells; PET: positron emission tomography; PI3K: phosphatidylinositol 3-Kinase; QoL: quality of life; Rb: retinobloastoma gene; SA: somatostatin analogue; SR: slow release; SST: somatostatin; SSTR: somatostatin receptor; STAT: signal transducers and activators of transcription; TACE: transarterial chemoembolisation; TGF: transforming growth factor; TNF: tumour necrosis factor; VEGF: vascular-endothelial growth factor; VLDL: very-low-density.

## Competing interests

A.C. and R.P. report to receive fee as consultants to Novartis, Ipsen and Pfizer and clinical grant supports. The remaining authors do not have any relationships to disclose.

## Authors’ contributions

CP contributed to the writing of the manuscript, MN, FC, GC, FI, AC contriduted to search the literature and to draft the manuscript, RP and MCDM contributed to the critical revision of the manuscript. All authors read and approved the final manuscript.
